# Functions and mechanisms of RNA tailing by metazoan terminal nucleotidyltransferases

**DOI:** 10.1002/wrna.1622

**Published:** 2020-07-22

**Authors:** Vladyslava Liudkovska, Andrzej Dziembowski

**Affiliations:** ^1^ Laboratory of RNA Biology International Institute of Molecular and Cell Biology Warsaw Poland; ^2^ Institute of Genetics and Biotechnology, Faculty of Biology University of Warsaw Warsaw Poland

**Keywords:** polyadenylation, TENT5, terminal nucleotidyltransferase, TUT4/7, uridylation

## Abstract

Termini often determine the fate of RNA molecules. In recent years, 3′ ends of almost all classes of RNA species have been shown to acquire nontemplated nucleotides that are added by terminal nucleotidyltransferases (TENTs). The best‐described role of 3′ tailing is the bulk polyadenylation of messenger RNAs in the cell nucleus that is catalyzed by canonical poly(A) polymerases (PAPs). However, many other enzymes that add adenosines, uridines, or even more complex combinations of nucleotides have recently been described. This review focuses on metazoan TENTs, which are either noncanonical PAPs or terminal uridylyltransferases with varying processivity. These enzymes regulate RNA stability and RNA functions and are crucial in early development, gamete production, and somatic tissues. TENTs regulate gene expression at the posttranscriptional level, participate in the maturation of many transcripts, and protect cells against viral invasion and the transposition of repetitive sequences.

This article is categorized under:

RNA Interactions with Proteins and Other Molecules > Protein‐RNA Recognition
RNA Processing > 3′ End Processing
RNA Turnover and Surveillance > Regulation of RNA Stability

## Graphical abstract

Terminal nucleotidyltransferases are a diverse group of enzymes that regulate many processes in metazoan cells. Here, we comprehensively summarize recent work on the role of posttranscriptional 3′ end RNA extensions in metabolism, organism development, and disease progression.

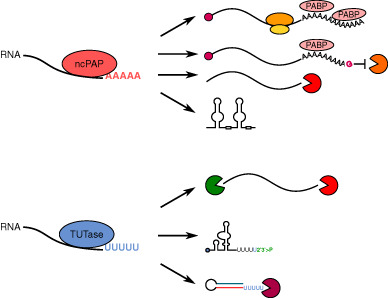

## 1 INTRODUCTION

Gene expression is precisely regulated at multiple levels to ensure the correct function of cells under normal conditions and rapidly and correctly respond to continually changing environmental conditions. A substantial part of such regulation is provided by tight coordination between transcriptional and posttranscriptional mechanisms. The posttranscriptional addition of nontemplated nucleotides to the 3′ end of various types of RNA molecules, also known as RNA tailing, is a highly conserved process that modulates their stability and activity in multiple organisms and plays a crucial role in regulating gene expression. The 3′ ends of RNA molecules, especially their tails, are a powerful regulatory location that can determine the fate of target RNAs. Almost all classes of RNA species can be subjected to some kind of 3′ end tailing at various steps of their existence. Thus, enzymes that are responsible for these extensions, namely terminal nucleotidyltransferases (TENTs), play an especially important role among RNA metabolism machinery.

**FIGURE 1:**
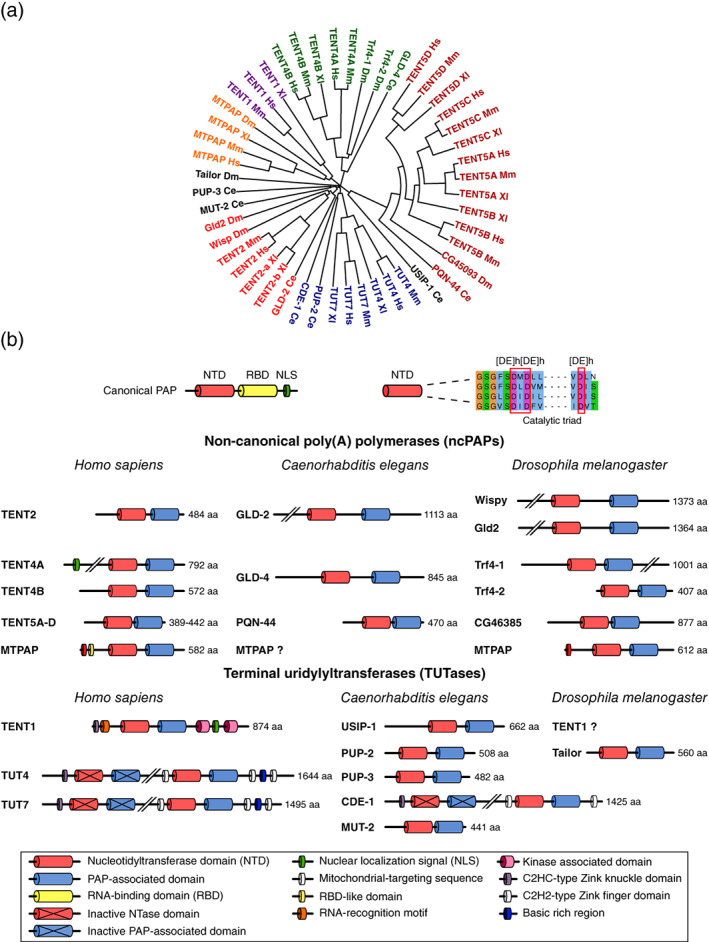
(a) A phylogenetic relationship of TENTs from vertebrates, worms, and fruit fly. (b) Schematic representation of terminal nucleotidyltransferases (TENTs) from the human, worm, and fruit fly genomes. Canonical nuclear poly(A) polymerase (PAP) is a highly evolutionarily conserved PAP. It comprises an N‐terminal nucleotidyltransferase (NTase) catalytic domain, a central RNA‐binding domain (RBD), and a C‐terminal domain with a nuclear localization signal (NLS). Catalytic activity relies on the highly conserved aspartate triad ([DE]h[DE]h [DE]h in the NTase domain. All TENTs share similar catalytic domain architecture but lack the RBD. The domain architecture of TENTs is diverse, and all additional domains and regions are indicated for each TENT. The length (aa) of each enzyme is indicated on the right

The best‐described role of 3′ tailing is the bulk polyadenylation of messenger RNAs (mRNAs) in the cell nucleus that is catalyzed by canonical poly(A) polymerases (PAPs), which also belong to the TENT superfamily (Aravind & Koonin, 1999; Martin & Keller, 2007). Canonical PAPs are, however, outside the scope of the present review, which focuses on other TENTs that play more specific regulatory roles. Various genomes encode different numbers of TENTs per organism. In humans, 11 TENTs are grouped into 6 subfamilies (TENT1–TENT6), based on their phylogenetic analysis (Figure 1a). The nomenclature of TENTs can be somewhat confusing because the same enzymes have often been described under different names. The updated nomenclature that was approved by the HUGO Gene Nomenclature Consortium advises referring to all vertebrate noncanonical PAPs (ncPAPs) and terminal uridylyltransferases (TUTases) by their respective TENT subfamily name. However, for some enzymes (e.g., mitochondrial poly(A) RNA polymerase [MTPAP], TUT4, and TUT7), their well‐recognized functional names can still be used. The invertebrate orthologs are named according to nomenclature guidelines for the respective organism, and their names do not always correspond to the human orthologs. To aid readers of the present review, Table 1 summarizes information about the nomenclature and functional diversity of TENTs in vertebrates, *Caenorhabditis elegans* and *Drosophila melanogaster*.

**TABLE 1:** Summary of current knowledge about metazoan TENTs

Subfamily	Enzyme (synonyms)	Activity	RNA substrate	Effect on target RNAs	Reference
*Vertebrates*					
TENT1	TENT1 (TUT1, U6 TUTase, Star‐PAP, PAPD2)	Oligouridylation	U6 snRNA	Maturation	Trippe et al. (2006)
		Polyadenylation (controversial)	Pre‐mRNA	Stabilization	Laishram and Anderson (2010), Mellman et al. (2008)
TENT2	TENT2 (GLD‐2, PAPD4, TUT‐2)	Polyadenylation	mRNA	Activation of translationally dormant mRNA	Barnard et al. (2004), Rouhana et al. (2005), Swanger et al. (2013), Udagawa et al. (2012), Yamagishi et al. (2016)
		Monoadenylation	miRNA	Stabilization	D'Ambrogio et al. (2012), Hojo et al. (2020), Katoh et al. (2009), Kim et al. (2016)
TENT3	TUT4 (TENT3A, PAPD3, ZCCHC11, KIAA0191) TUT7 (TENT3B, PAPD6, ZCCHC6, KIAA1711)	Oligouridylation	mRNA Histone mRNA LINE‐1 mRNA Pre‐miRNA miRNA Pre‐rRNA Pol III‐ncRNAs Viral RNA TSS RNA	Degradation	Chang et al. (2014, 2018), Faehnle et al. (2014), Gutiérrez‐Vázquez et al. (2017), Heo et al. (2009), Jones et al. (2012), Łabno et al. (2016), Lackey et al. (2016), Le Pen et al. (2018), Lim et al. (2014), Morgan et al. (2019, 2017), Mullen and Marzluff (2008), Pirouz et al. (2016, 2019), Piskounova et al. (2011), Thornton et al. (2014), Ustianenko et al. (2018, 2013, 2016), Warkocki, Krawczyk, et al. (2018)
		Monouridylation	Pre‐miRNA	Processing	Heo et al. (2012), Kim et al. (2015)
TENT4	TENT4A (PAPD7, POLS, TRF4‐1, TUT5, POLK)	Polyadenylation (tethering assay)	mRNA	Not defined	Ogami et al. (2013)
		Mixed A/G tailing (with TENT4B)	mRNA Viral RNA	Stabilization	Hyrina et al. (2019), Kim et al. (2020), Lim et al. (2018), Mueller et al. (2019)
	TENT4B (PAPD5, TRF4‐2, GLD‐4, TUT3)	Polyadenylation	Pre‐rRNA rRNA Y RNA hTR	Degradation	Fasken et al. (2011), Lubas et al. (2011), Preker et al. (2011), Shcherbik et al. (2010), Sinturel et al. (2017), Sudo et al. (2016)
			mRNA	Stabilization	Burns et al. (2011), Shin et al. (2017)
		Oligoadenylation	snoRNA scaRNA miRNA	Maturation Degradation	Berndt et al. (2012), Son et al. (2018) Shukla et al. (2019)
		Monoadenylation	miRNA	Degradation	(Boele et al. (2014)
		Mixed A/G tailing (with TENT4A)	mRNA Viral RNA	Stabilization	Hyrina et al. (2019), Kim et al. (2020), Lim et al. (2018), Mueller et al. (2019)
TENT5	TENT5A (FAM46A) TENT5B (FAM46B) TENT5C (FAM46C) TENT5D (FAM46D)	Polyadenylation	mRNA	Stabilization (shown for TENT5C and TENT5B)	Bilska et al. (2020), Herrero et al. (2020), Hu et al. (2020), Mroczek et al. (2012)
TENT6	MTPAP (TENT6, PAPD1, SPAX4)	Polyadenylation	Mt‐mRNA	Stop codon generation, stabilization	Anderson et al. (1981), Nagaike et al. (2005), Tomecki et al. (2004), Wilson et al. (2014)
		Oligoadenylation	Mt‐tRNA	Maturation	Fiedler et al. (2015), Pearce et al. (2017)
*Caenorhabditis elegans*					
TENT1	USIP‐1	Oligouridylation	U6 snRNA	Stabilization Recycling	Rüegger et al. (2015)
TENT2	GLD‐2	Polyadenylation	Oocyte mRNA	Activation Stabilization	Kim et al. (2010), Nousch et al. (2014), Schmid et al. (2009), Suh et al. (2006)
TENT3	CDE‐1 (PUP‐1, CID‐1)	Uridylation	siRNA Viral RNA	Degradation	Le Pen et al. (2018), van Wolfswinkel et al. (2009)
	PUP‐2	Uridylation	Pre‐miRNA	Processing	Lehrbach et al. (2009)
	PUP‐3	Uridylation			Preston et al. (2019)
	MUT‐2 (RDE‐3)	Tandem UG repeats addition	siRNA Transposons	Trigger gene silencing	Preston et al. (2019), Shukla et al. (2020)
TENT4	GLD‐4	Polyadenylation	Oocyte mRNA	Not defined	Nousch et al. (2014)
TENT5	PQN‐44 (TENT5)	Putative ncPAP			
*Drosophila melanogaster*					
TENT2	Gld2	Polyadenylation	mRNA	Activation	Jae et al. (2008), Sartain et al. (2011)
	Wispy	Polyadenylation	mRNA	Activation Stabilization	Benoit et al. (2008), Coll et al. (2018), Cui et al. (2008, 2013), Dufourt et al. (2017), Eichhorn et al. (2016), Lim et al. (2016)
		Oligoadenylation	miRNA sisRNAs	Degradation Stabilization	Lee et al. (2014), Jia Ng et al. (2018)
TENT3	Tailor	Oligouridylation	Mirtrons 5S rRNA tRNA snRNA snoRNA RNase MRP	Degradation	Bortolamiol‐Becet et al. (2015), Reimão‐Pinto et al. (2015), Reimão‐Pinto et al. (2016)
TENT4	Trf4‐1	Polyadenylation	snRNA mRNA	Degradation	Harnisch et al. (2016), Nakamura et al. (2008)
	Trf4‐2	Inactive			Nakamura et al. (2008)
TENT5	CG46385	Putative ncPAP			

Both TENTs and canonical PAPs belong to the nucleotidyltransferase superfamily of DNA polymerase β, which is conserved in eukaryotes. Canonical PAP comprises three domains: an N‐terminal nucleotidyltransferase (NTase) catalytic domain with the signature helix‐turn motif, a central domain, and a C‐terminal RNA‐binding domain (RBD) with a nuclear localization signal and multiple regulatory sites (Martin, Keller, & Doublié, 2000; Yang, Nausch, Martin, Keller, & Doublié, 2014). Three conserved aspartates in the NTase domain are essential for catalytic activity. TENTs share a similar catalytic domain architecture and the mechanism of catalysis with canonical PAP, but they also have some notable differences (Figure 1b). TENTs carry the conserved NTase domain but lack a typical RBD (with the exception of TENT1). The NTase domain of TENTs is accompanied by a PAP‐associated domain that is absent in canonical PAP. TENTs could also carry some additional regulatory elements, such as sequences that are responsible for subcellular localization or accessory domains to perform their diverse functions (Martin & Keller, 2007). Based on their preference for adenosine triphosphate (ATP) or uridine triphosphate (UTP) incorporation, TENTs are divided into ncPAPs and TUTases. However, some TENTs may exhibit broader specificity for the incorporated ribonucleotide, depending on the cellular context, and can incorporate more than one type of ribonucleotide, thereby leading to so‐called mixed tailing.

Despite the fact that all TENTs belong to one superfamily and share a conserved catalytic domain, they have remarkable functional diversity that affects RNA maturation, stability, and decay and the activation of translationally dormant deadenylated mRNAs (Martin, Doublié, & Keller, 2008). Moreover, TENTs from different organisms have been shown to play specific regulatory roles in the nucleus, cytoplasm, and mitochondria. Finally, TENTs add mono‐, oligo‐, and polynucleotides that are homo‐ or heteroribonucleotide extensions to a variety of RNA substrates, including mRNA, small nuclear RNA (snRNA), micro RNA (miRNA), and aberrant rRNA. Such variables (i.e., substrates, localization, added extensions, and functional outcome) make RNA tailing a potent instrument of the posttranscriptional regulation of gene expression. In recent years, many groundbreaking discoveries of the role of TENTs in many important processes in different organisms have been made, and the development of new high‐throughput methods (Box 1) has allowed our understanding of RNA 3′ end modifications to substantially expand. Many excellent review articles have described the role of mammalian TENTs in the regulation of gene expression (De Almeida, Scheer, Zuber, & Gagliardi, 2018; Menezes, Balzeau, & Hagan, 2018; Warkocki, Liudkovska, Gewartowska, Mroczek, & Dziembowski, 2018; Yu & Kim, 2020). However, these enzymes also act in other organisms. Many crucial findings have been reported using invertebrate model organisms, the most studied of which are *C. elegans* and *D. melanogaster*.

BOX 1 **HIGH‐THROUGHPUT METHODS TO STUDY RNA 3′ END EXTENSIONS**

*TAIL‐seq* allows researchers to determine the exact 3′ end of RNA molecules, measure the poly(A) tail length, and capture noncanonical tail modifications, such as uridylation and guanylation (Chang, Lim, Ha, & Kim, 2014). To prepare sequencing libraries, total RNA is subjected to the size‐fractionation (>200 nt) and depletion of ribosomal RNA (rRNA). Next, the biotinylated 3′ adaptor is ligated, and RNA is treated with a low concentration of RNase T1, which partially digests the RNA body but leaves poly(A) tails intact. The fragmented RNA is pulled down with streptavidin beads, phosphorylated at the 5′ end, and gel purified (500–1,000 nt). Following ligation of the 5′ adaptor, RNA is reverse transcribed, amplified by polymerase chain reaction (PCR), and sequenced in the pair‐end mode. A dedicated algorithm is used to detect signals from long thymine stretches (Chang et al., 2014). TAIL‐seq is a powerful method, but it is technically challenging and expensive. In a modified version of TAIL‐seq (mRNA TAIL‐seq [*mTAIL‐seq*]), splint ligation of the 3′ hairpin adaptor is used to significantly increase sensitivity of the method to mRNAs and decrease costs (Lim, Lee, Son, Chang, & Kim, 2016). The main drawback of mTAIL‐seq is its limited ability to capture very short tails (<8 nt) and tails with non‐adenosine terminal nucleotides. Although enrichment for specific types of the RNA terminus is possible by changing the design of splint ligation adaptors, mTAIL‐seq is still less effective for comprehensively studying all types of RNA termini than the original method and is mostly used to analyze the poly(A) tail length (Lim et al., 2016).

*PAL‐seq* (poly(A) tail length profiling by sequencing) accurately measures the length of poly(A) tails (Subtelny, Eichhorn, Chen, Sive, & Bartel, 2014). A splint 3′ adaptor is specifically ligated to 3′ ends of polyadenylated RNA after partial digestion with RNase T1. The gel‐purified mRNA fragments are bound to streptavidin beads before phosphorylating the 5′ ends for adaptor ligation, reverse transcribed, and released from the beads. The sequencing clusters are generated by annealing the primers to the 3′ end of the poly(A) sequence and extended using deoxythymidine triphosphate and biotinylated deoxyuridine triphosphate (dUTP). Next, fluorescent streptavidin tags are attached to biotin‐dUTP, and their signal intensity is used to quantify the length of adenosine homopolymers. One of the main limitations of PAL‐seq is that it offers information about RNA 3′ ends that consist only of adenosines and does not reveal non‐adenosine tailings. PAL‐seq also requires a highly elaborate and customized setup that can be executed only on the discontinued Illumina Genome Analyzer II sequencer. The newest version of the method, PAL‐seq v2, combines the advantages of TAIL‐seq and PAL‐seq and allows researchers to measure poly(A) tails with non‐adenosine termini using more readily accessible contemporary Illumina sequencing equipment. For further technical details, see Eisen et al. (2020).

*FLAM‐seq* (full‐length poly(A) and mRNA sequencing) and *PAIso‐seq* (poly(A) inclusive RNA isoform sequencing) enable the global and accurate determination of the full‐length sequence of endogenous mRNAs, including their poly(A) tails along with non‐adenosine residues at all positions within the tails (Legnini, Alles, Karaiskos, Ayoub, & Rajewsky, 2019; Liu, Nie, Liu, & Lu, 2019). The principle of both methods is very similar and includes an RNA 3′ end extension, template‐switching, cDNA amplification from full‐length mRNA, and PCR amplification following PacBio sequencing. The main difference between FLAM‐seq and PAIso‐seq lies in the approach to add a 3′ adaptor to the end of poly(A) tails. FLAM‐seq uses G/I tailing, whereas PAIso‐seq employs template extension. FLAM‐seq and PAIso‐seq have comparable accuracy, but PAIso‐seq is possibly more sensitive and suitable for single‐cell analysis.

*Oxford nanopore direct RNA sequencing* allows long‐read full RNA molecule sequencing and accurate quantification of the poly(A) tail length without a need for RNA fragmentation or amplification, thus significantly reducing bias that can be introduced by the PCR amplification of long adenosine tracts within tails (for review, see Feng, Zhang, Ying, Wang, & Du, 2015). The 3′ sequencing primer that carries the motor protein is attached to the RNA molecule or RNA–DNA hybrid after optional reverse transcription. The motor protein passes the RNA strand through the pore at a consistent rate and in an ATP‐dependent manner. Sequencing then proceeds in the 3′–5′ direction, starting from the adaptor primer. Although this technology is one of the best for analyzing the poly(A) tail length, it does not yet allow the identification of non‐adenosine tail insertions or tailing. Moreover, a large amount of purified mRNA is currently still required for this method.

*TRAID‐seq* (tethered rNTase activity identified by high‐throughput sequencing) is a screening approach to analyze nontemplated 3′ end extensions that are added to reporter RNA by a tethered known or putative TENT (Preston et al., 2019). TRAID‐seq allows both the assessment of candidate enzyme activity and single‐nucleotide resolution analysis of the identity of the added tailing. A specific TENT is fused to MS2 coat protein and expressed in yeast together with a reporter RNA that carries high‐affinity MS2‐binding sites. The interaction between MS2 and its binding site in the reporter brings the fused TENT to the reporter RNA, and its 3′ end modifications are then assessed by Illumina sequencing.

## 2 NONCANONICAL POLYADENYLATION

The role of poly(A) tails that are added by canonical nuclear PAP is well‐described. They are crucial for regulating mRNA stability, export to the cytoplasm, the regulation of mRNA translation, and decay. Noncanonical nuclear and cytoplasmic polyadenylation may have varying effects on target RNA molecules, depending on the particular enzyme or subcellular localization. This section summarizes our current knowledge about all metazoan families of ncPAPs.

### 2.1 TENT2, GLD‐2, Wispy, and dmGLD2

Germline development defective‐2 (GLD‐2) was first identified in *C. elegans* as a gene that controls the germline mitosis‐to‐meiosis decision, progression through meiosis, and gamete production (Kadyk & Kimble, 1998; Wang, Eckmann, Kadyk, Wickens, & Kimble, 2002). GLD‐2 is evolutionary conserved, and its homologs are also active PAPs in other species, including *D. melanogaster*, *Xenopus laevis*, and mammals (Barnard, Ryan, Manley, & Richter, 2004; Benoit, Papin, Kwak, Wickens, & Simonelig, 2008; Cui, Sackton, Horner, Kumar, & Wolfner, 2008; Kwak, Wang, Ballantyne, Kimble, & Wickens, 2004; Rouhana et al., 2005; Sartain, Cui, Meisel, & Wolfner, 2011). Similar to the majority of all TENTs, GLD‐2/TENT2 lacks the RNA‐recognition motif and is recruited to its various substrate RNAs by other RNA‐binding proteins. In vitro, GLD‐2 itself has low catalytic activity; in the presence of its co‐factors, however, it can effectively elongate mRNA tails (Wang et al., 2002). At least in worms, all known molecular and biological functions of GLD‐2 may depend on its enzymatic activity (Nousch, Minasaki, & Eckmann, 2017).

**FIGURE 2:**
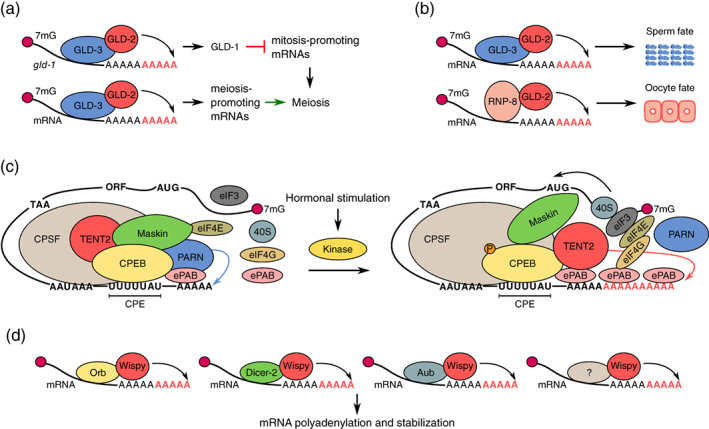
Selected aspects of polyadenylation by GLD‐2, TENT2, and Wispy in worms, frogs, and flies, respectively. (a) Polyadenylation by GLD‐2/GLD‐3 plays an important role in the mitosis‐to‐meiosis decision in the adult worm germline through the stabilization of *gld‐1* mRNA, which encodes a repressor of mitosis‐promoting mRNAs. At the same time, GLD‐2/GLD‐3 activates repressed meiosis‐related mRNAs. Both events result in the switch from mitosis to meiosis. (b) In worms, GLD‐2 forms two separate complexes with RNA‐binding proteins. GLD‐2/GLD‐3 specifies spermatogenesis, and GLD‐2/RNP‐8 specifies oogenesis. (c) One of the proposed mechanisms of mRNA reactivation in *Xenopus* oocytes. In immature *Xenopus* oocytes, CPE‐containing mRNAs (UUUUUAU motif) are bound by CPEB and form a complex with CPSF, TENT2, PARN, ePABP, and Maskin that prevents assembly of the translation initiation complex and thus keeps mRNAs translationally repressed. Upon hormonal stimulation, CPEB is phosphorylated that leads to substantial rearrangements. PARN dissociates from the complex and allows TENT2 to extend poly(A) tails. As a result, ePABP binds to the newly elongated tails. eIF4G displaces Maskin from eIF4E and promotes formation of the translation initiation complex, initiating the translation of mRNAs. 40S, small subunit of the cytoplasmic ribosome; CPE, cytoplasmic polyadenylation element; CPEB, CPE‐binding protein; CPSF, cleavage and polyadenylation specificity factor; eIF3, eukaryotic initiation factor 3; eIF4, eukaryotic initiation factor 4; ePABP, embryonic poly(A) binding protein; PARN, poly(A)‐specific ribonuclease. (d) In the fruit fly, Wispy is recruited to its target mRNAs via interactions with various RNA‐binding proteins

In *C. elegans*, GLD‐2 influences multiple aspects of germline development. High‐throughput analysis and case‐specific studies identified over 500 germline‐enriched mRNAs that are potential substrates for GLD‐2, the expression levels of which are significantly downregulated in *gld‐2*‐defective worms (Kim, Wilson, & Kimble, 2010; Nousch, Yeroslaviz, Habermann, & Eckmann, 2014; Schmid, Küchler, & Eckmann, 2009; Suh, Jedamzik, Eckmann, Wickens, & Kimble, 2006). Although some of these mRNAs may have been indirectly downregulated because of the developmental defect of *gld‐2* mutants, the number of direct GLD‐2 substrates was still very high. The loss of *gld‐2* led to the shortening of mRNA poly(A) tails and a decrease in mRNA abundance, suggesting that GLD‐2 promotes the stability and expression of translationally repressed germline mRNAs (Kim et al., 2010; Nousch et al., 2014). GLD‐2, together with its cofactor GLD‐3 (an RNA‐binding protein of the Bicaudal‐C family), plays an important role in entry and progression through the meiosis of germ cells (Figure 2a) (Eckmann, Crittenden, Suh, & Kimble, 2004). One of the critical targets of GLD‐2 is *gld‐1* mRNA that encodes the repressor of mitosis‐promoting mRNAs (Suh et al., 2006). Translationally repressed *gld‐1* mRNA ensures mitosis in the germline (for review, see Kimble & Crittenden, 2007; Wang & Voronina, 2020). However, upon the GLD‐2/GLD‐3‐mediated polyadenylation of *gld‐1* mRNA, newly translated GLD‐1 protein promotes the germ cell mitosis‐to‐meiosis transition by repressing mitosis‐specific mRNAs. Moreover, GLD‐2 may also activate other mRNAs that are required for meiosis. Other processes that are regulated by GLD‐2 include gamete identity determination, progression through spermatogenesis and oogenesis, and oocyte development (Kim et al., 2009, 2010). These functions of GLD‐2 are facilitated by its selective interactions with two distinct RNA‐binding cofactors, RRM domain‐containing protein 8 (RNP‐8) and GLD‐3 (Figure 2b). During gamete production in hermaphrodites, GLD‐3 and RNP‐8 compete with each other and form separate complexes with GLD‐2 to promote its activity toward distinct pools of mRNA targets (Eckmann et al., 2004; Kim et al., 2009, 2010; Nakel et al., 2016; Nakel, Bonneau, Eckmann, & Conti, 2015). Gamete production in worms starts with spermatogenesis and later switches to oogenesis. Through differential interactions with GLD‐3 and RNP‐8, GLD‐2 controls gamete sex specification. GLD‐2/GLD‐3 promotes spermatogenesis, whereas GLD‐2/RNP‐8 specifies oogenesis (Kim et al., 2009; Nakel et al., 2016). Interestingly, instead of antagonizing each other, RNP‐8 and GLD‐3 have been shown to act synergistically during oocyte development (Kim et al., 2010).

The role of TENT2/GLD‐2 in gamete production is conserved among many species. Cytoplasmic polyadenylation has been studied in great detail throughout oocyte maturation and early embryo development in the frog *X. laevis* (for a comprehensive review, see Charlesworth, Meijer, & De Moor, 2013; Ivshina, Lasko, & Richter, 2014; Reyes & Ross, 2016). In frog oocytes that are arrested at the end of the first meiotic prophase, numerous maternal mRNAs are stored with short poly(A) tails to prevent their translation. These mRNAs acquire their dormant state through the association of their U‐rich cytoplasmic polyadenylation element (CPE) that is located in 3′ untranslated regions with CPE‐binding protein (CPEB). mRNA‐CPEB further interacts with cleavage and polyadenylation specificity factor, and the whole complex is exported to the cytoplasm. Two models have been proposed for the mechanism of the CPEB‐mediated inhibition of mRNA translation and its subsequent activation (for review, see Kang & Han, 2011; Radford, Meijer, & de Moor, 2008). However, both require the activity of TENT2 for mRNA readenylation after hormonal stimulation of the oocytes. In the first model, CPEB recruits the poly(A)‐specific ribonuclease PARN which competes with TENT2 activity to keep poly(A) tails short. Additionally, CPBE interacts with Maskin, which binds cap‐binding protein eukaryotic initiation factor 4E (eIF4E) and prevents translation. The phosphorylation of CPEB upon the hormonal stimulation of oocytes causes the dissociation of PARN, elongation of the poly(A) tail by TENT2, and consequently the release of Maskin from the masked mRNA (Figure 2c). Notably, however, the mammalian ortholog of Maskin, known as transforming acidic coiled‐coil‐containing protein 3 (TACC3), is a nonmotor microtubule‐associated protein that plays an important role in mitotic spindle stability and organization (O'Brien et al., 2005; Yao, Natsume, & Noda, 2007). The second model of CPEB action does not require Maskin, which interaction with CPEB was shown to be weak and probably not conserved (Minshall, Reiter, Weil, & Standart, 2007). Instead, CPEB is proposed to recruit the RNA helicase Xp54, RNA‐associated protein 55 (RAP55), and 4E‐T protein to interact with eIF4E1b. The presence of this complex is proposed to stop the translation of targeted mRNAs (Minshall et al., 2007).

The *D. melanogaster* genome encodes two homologs of TENT2/GLD‐2. The first homolog, Gld2 (CG5732), is highly enriched in the testes and acts during postmeiotic spermatogenesis (Sartain et al., 2011). Gld2‐deficient males do not produce mature sperm and are sterile (Sartain et al., 2011). Consistent with its role in fly spermatogenesis, Gld2 was found to interact with the GLD‐3 homolog BIC‐C in a yeast two‐hybrid assay (Sartain et al., 2011). Surprisingly, the enzymatic activity of Gld2 is also essential for long‐term memory formation in neurons (Jae et al., 2008). The second homolog, Wispy (CG15737), is expressed in the female germline and is required for fruit fly oogenesis (Benoit et al., 2008; Cui et al., 2008; Cui, Sartain, Pleiss, & Wolfner, 2013). Wispy polyadenylates the vast majority of maternal mRNAs, with the exception of ribosomal protein transcripts, during late oogenesis and upon egg activation (Benoit et al., 2008; Cui et al., 2008, 2013; Eichhorn et al., 2016; Lim et al., 2016). Interestingly, Wispy may not only activate these mRNAs but also protect their poly(A) tails from deadenylation. In *wispy* mutants, poly(A) tail lengths are shortened during late oogenesis (Lim et al., 2016). Although the homolog of CPEB, Orb, interacts with Wispy (Benoit et al., 2008), not all maternal mRNAs carry Orb‐binding sequences, suggesting that Orb is not the only regulator of cytoplasmic polyadenylation during oogenesis in flies (Figure 2d) (Cui et al., 2013; Ivshina et al., 2014). Indeed, Wispy polyadenylates the innate immunity mRNA *Toll* in a Dicer‐2‐dependent manner. Dicer‐2 is implicated in RNA interference (RNAi), but its role in cytoplasmic polyadenylation is independent of RNAi machinery (Coll et al., 2018). The PIWI protein Aubergine also acts as a Wispy‐recruiting factor to stabilize mRNAs in the *Drosophila* germ plasm (Dufourt et al., 2017). Wispy polyadenylates and stabilizes so‐called stable intronic sequence RNAs (sisRNAs) during fly oocyte development (Jia Ng, Zheng, Osman, & Pek, 2018). sisRNAs are long intronic sequences that are excised during splicing and present as extremely stable linear or lariat molecules with high regulatory potential (for review, see Chan & Pek, 2019). The exact function of sisRNAs in fruit flies is only beginning to be known, but some have been proposed to be required for proper fly larva development via the clearance of long noncoding RNAs (Jia Ng et al., 2018). The mechanism of Wispy recruitment to sisRNAs remains to be established.

In contrast to invertebrates and *Xenopus*, the involvement of mammalian TENT2 in gametogenesis and early embryo development is still not precisely known, since the disruption of the *TENT2* gene has no effect on poly(A) tail elongation in mouse oocytes or on oocyte maturation (Nakanishi et al., 2007). Furthermore, TENT2‐deficient male and female mice are fertile and healthy (Nakanishi et al., 2007), raising the possibility that other TENTs might be involved in mRNA polyadenylation in the mammalian germline (Kashiwabara et al., 2002). TENT2 function has been better described in the mouse brain. TENT2 is ubiquitously expressed in the cerebellum and hippocampus and proposed to regulate synaptic plasticity (Rouhana et al., 2005). TENT2 activates over 100 mRNAs in mouse hippocampal dendrites in a mode that is similar to frog oocytes, in which synaptic stimulation induces the phosphorylation of CPEB, leading to protein rearrangement on masked mRNAs and the polyadenylation of these mRNAs by TENT2 (Udagawa et al., 2012). Among the most prominent targets of TENT2 in the hippocampus are mRNAs that encode the NR2A and GluN2A subunits of *N*‐methyl‐d‐aspartate receptors that are necessary for long‐term synaptic plasticity (Swanger, He, Richter, & Bassell, 2013; Udagawa et al., 2012). TENT2 knockout animals do not exhibit any behavioral abnormalities.

In somatic cells, other RNA‐binding proteins may recruit TENT2 to target mRNAs for polyadenylation and the activation of translation. For example, QKI‐7 specifically targets heterogeneous nuclear ribonucleoprotein A1 (hnRNPA1), p27, and β‐catenin mRNAs for TENT2 polyadenylation in human embryonic kidney 293 (HEK293) cells (Yamagishi, Tsusaka, Mitsunaga, Maehata, & Hoshino, 2016).

In addition to their role in mRNA expression, TENT2 and Wispy have been implicated in regulating the stability of certain mature miRNAs. *TENT2* gene deletion in a human fibroblast cell line significantly reduced a fraction of monoadenylated miRNAs (D'Ambrogio, Gu, Udagawa, Mello, & Richter, 2012). The liver‐specific miRNA‐122 plays an essential role in liver development and metabolism and has been shown to be 3′ monoadenylated in both human hepatocytes and the mouse liver (Katoh et al., 2009). The level of miRNA‐122 is significantly lower in TENT2 knockout mice, suggesting that TENT2 monoadenylation enhances its stability (Katoh et al., 2009). Moreover, miRNA‐122 stabilization in hepatocytes is promoted by QKI‐7. Similar to HEK293 cells, QKI‐7 in hepatocytes recruits TENT2 for miRNA‐122 monoadenylation (Hojo et al., 2020). Another example of a TENT2‐stabilizing effect on miRNA‐122 was the observation that the hepatitis C virus core protein promotes miRNA‐122 decay by inhibiting TENT2 (Kim et al., 2016). Interestingly, the depletion of TENT2 adenylation has also been shown to did not affect miRNA stability (Burroughs et al., 2010; Mansur et al., 2016). Furthermore, an opposite effect of maternal miRNA adenylation was reported in fly early embryos (Lee et al., 2014). The deletion of *wispy* resulted in the accumulation of miRNAs, whereas its overexpression increased adenylation and reduced miRNA abundance, suggesting that Wispy‐mediated adenylation may promote miRNA decay and clearance during the maternal‐to‐zygotic transition. miRNA adenylation is proposed to be mediated by an interaction between Wispy and Argonaute 1, presenting yet another selective recruiting factor (Lee et al., 2014). Still unclear is what exactly specifies the adenylation effect on miRNA stability. One speculation is that the addition of one adenosine may stabilize miRNA, whereas a longer oligo(A) tail may recruit exonucleases and result in miRNA degradation. The processivity of TENT2 and adenylation effect on miRNA stability may also depend on the cell type, molecular context, and particular miRNA species.

### 2.2 TENT4A, TENT4B, GLD‐4, and Trf4‐1

**FIGURE 3:**
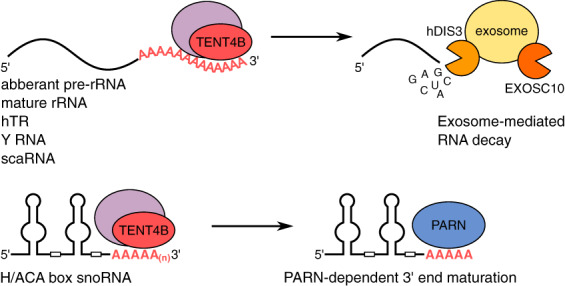
In the nucleus, polyadenylation by TENT4B/A, acting alone or in complex with other proteins, induces the exosome‐mediated decay of various RNA species (upper panel). TENT4B/A also cooperates with the poly(A)‐specific ribonuclease PARN to promote H/ACA box snoRNA maturation (bottom panel)

Human TENT4A and TENT4B are orthologs of the yeast ncPAPs Trf4p and Trf5p (Houseley & Tollervey, 2006; San Paolo et al., 2009). In yeast, Trf4p, together with the zinc knuckle protein Air2p and RNA helicase Mtr4p, forms the Trf4/Air2/Mtr4p polyadenylation (TRAMP) complex, which recruits the nuclear exosome to degrade various RNA species (LaCava et al., 2005; Vaňáčová et al., 2005). TENT4A and TENT4B have a high level of similarity, but there is only partial overlap between their described functions (Burroughs et al., 2010; Lim et al., 2018; Lubas et al., 2011; Shcherbik, Wang, Lapik, Srivastava, & Pestov, 2010). TENT4A exists in two isoforms that differ in their N‐terminal region: TENT4A short (S) and the more prevalent TENT4A long (L) (Lim et al., 2018; Ogami, Cho, & Hoshino, 2013). Only TENT4A (L) can polyadenylate reporter mRNA in the tethering assay. TENT4A (L) localizes predominantly in the nucleus, and its unique N‐terminal region is crucial for the nuclear localization and activity. In contrast, inactive TENT4A (S) is distributed evenly in the cell (Ogami et al., 2013). The functions of TENT4A have not been extensively studied. TENT4A has not been shown to be a component of the human TRAMP complex, and unknown is whether it can form TRAMP‐like complexes (Fasken et al., 2011). The functional relevance of TENT4B is much closer to its yeast ortholog (Figure 3). TENT4B localizes to the nucleus, accumulates in the nucleolus, and forms a conserved TRAMP complex with the Air1/Air2p homolog ZCCHC7 and Mtr4p homolog SKIV2L2 (Fasken et al., 2011; LaCava et al., 2005; Lubas et al., 2011; Rammelt, Bilen, Zavolan, & Keller, 2011; Sudo, Nozaki, Uno, Ishida, & Nagahama, 2016; Weick et al., 2018). As a part of the TRAMP complex, TENT4B adds tails and promotes either the decay or maturation of various RNA species.

The role of TENT4B/Trf4p in rRNA surveillance is highly conserved between yeast and mammals (Dez, Houseley, & Tollervey, 2006; LaCava et al., 2005). TENT4B participates in the adenylation of aberrant and superfluous precursor rRNA (pre‐rRNA) fragments, leading to their degradation by the nucleolar exosome (Lubas et al., 2011; Shcherbik et al., 2010; Sudo et al., 2016). Additionally, TENT4B‐mediated polyadenylation contributes to the degradation of mature rRNAs in mouse hepatocytes. In mice, daily oscillations in liver mass and size result from regulated changes in the number and activity of ribosomes (Sinturel et al., 2017). During a diurnal cycle, pre‐rRNAs are continually synthesized, whereas ribosomal proteins are produced rhythmically, leading to a situation in which not all rRNAs are packed into mature ribosomes. Excess 18S and 28S rRNAs that are not incorporated into ribosomes are polyadenylated by TENT4B and degraded by the nuclear exosome (Sinturel et al., 2017). Another conserved function of TENT4B as part of the TRAMP complex involves formation of the mature 3′ end of small nucleolar RNAs (snoRNAs) (Grzechnik & Kufel, 2008; LaCava et al., 2005). In humans, TENT4B participates in the maturation of H/ACA box snoRNAs by adding oligo(A) tails to the processing intermediate after initial 3′–5′ exonucleolytic trimming of the excised intron that contains the snoRNA precursor (Figure 3). The oligo(A) tails, together with remaining intronic nucleotides of snoRNA, are then trimmed by PARN, which generates mature and stable snoRNAs (Berndt et al., 2012; Son, Park, & Kim, 2018).

The adenylation by TENT4B may influence the targeting of some miRNAs and, together with PARN and other 3′–5′ exoribonucleases, affect their stability (Burroughs et al., 2010; Rammelt et al., 2011; Shukla, Bjerke, Muhlrad, Yi, & Parker, 2019; Wyman et al., 2011). Similar to the TENT2‐mediated adenylation of miRNA, adenylation by TENT4 may have either a positive or negative impact on miRNA stability that likely depends on the cellular context and length of the added tail. Monoadenylation of the 3′ end of oncogenic miRNA‐21 marks it for direct degradation by PARN (Boele et al., 2014). Intriguingly, this observation is opposite to the monoadenylation effect of TENT2 that instead stabilizes miRNAs (D'Ambrogio et al., 2012; Hojo et al., 2020; Katoh et al., 2009). In a more complicated pathway, the oligoadenylation of miRNA‐25 and miRNA‐92 by TENT4B poises regulation of their stability to enable the tight and rapid modulation of their levels (Shukla et al., 2019). In such a scenario, PARN removes oligo(A) tails that protect these miRNAs from complete degradation by the 3′–5′ exonucleases DIS3‐like exonuclease 1 (DIS3L) and DIS3L2. As a result, these two ribonuclease activities of PARN versus DIS3L/DIS3L2 have an opposing outcome with regard to levels of miRNA that are sensitized by TENT4B (Shukla et al., 2019). Similar competition for the TENT4B‐oligoadenylated substrate between PARN and other exoribonucleases has been described for the human telomerase RNA (hTR) precursor (Roake et al., 2019; Shukla, Schmidt, Goldfarb, Cech, & Parker, 2016). The telomerase ribonucleoprotein component Dyskerin binds and stabilizes the hTR precursor, thereby promoting its maturation by the exosome. Dyskerin also controls mature hTR levels by recruiting TENT4B and PARN with the accumulation of oligoadenylated hTR that leads to its exosome‐mediated degradation (Roake et al., 2019; Shukla et al., 2016). TENT4B is also implicated in the removal of abundant RNA polymerase III (Pol III)‐transcribed Y RNAs (Shukla & Parker, 2017; Son et al., 2018). In humans, Y RNAs play a role in DNA replication, the DNA damage response, histone mRNA processing, and RNA quality control (Kowalski & Krude, 2015). Again, PARN can remove oligo(A) tails that are added by TENT4B, leading to the stabilization of these RNAs that are otherwise completely degraded by DIS3L (Shukla & Parker, 2017; Son et al., 2018). Finally, in a similar scenario, PARN and target of EGR1 protein 1 have also been shown to promote the maturation of small Cajal body‐specific RNAs by trimming their 3′ ends that contain oligo(A) tails that are added by TENT4B (Son et al., 2018). In summary, in all of the aforementioned cases, adenylation by TENT4B sensitizes the RNA molecule for both PARN and exosome activities but with opposing outcomes on its fate (i.e., stabilization and degradation, respectively). Such a mechanism probably allows better regulation of the levels of different RNA species.

Intriguingly, TENT4B may have a positive regulatory impact on mRNAs. Polyadenylation by TENT4B has been shown to stabilize *TP53* mRNA (also known as p53) and modulate its translational competence. In the proposed model, TENT4B is recruited to its target mRNA through its interaction with CPEB (Burns, D'Ambrogio, Nottrott, & Richter, 2011). TENT4B may also mediate the CPEB‐dependent stabilization of several mRNAs that are involved in carbohydrate metabolism, including *GLUT1* mRNA, which encodes the major glucose transporter (Shin, Paek, Ivshina, Stackpole, & Richter, 2017). However, other studies do not provide evidence of TENT4B‐CPEB interactions.

**FIGURE 4:**
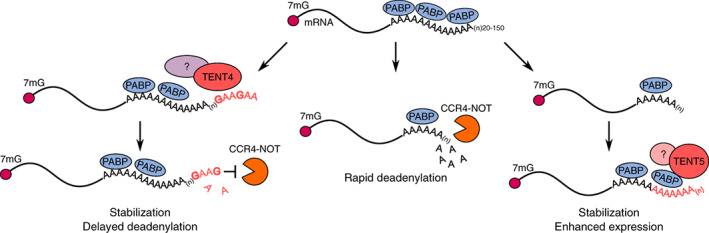
Stabilization of polyadenylated mRNAs. During their lifetime, the mRNA poly(A) tails become gradually shortened through CCR4‐NOT‐mediated deadenylation (center) that later leads to complete mRNA degradation from both ends (not shown). TENT4A/B enhances the stability of mRNAs by the mixed A/G tailing of their poly(A) tails (on the left). The guanosine residues in the terminal positions of poly(A) tails protect mRNA from deadenylation by CCR4‐NOT because CCR4‐NOT is unable to efficiently remove the guanidine residue. Proteins from the TENT5 family elongate mRNA poly(A) tails in the cytoplasm, ultimately extending the mRNA lifespan and enhancing its expression (on the right). Remaining unknown are the ways in which TENT4 and TENT5 ncPAPs recognize their target RNA and whether other protein cofactors facilitate their function

Another function that has been attributed to both TENT4B and TENT4A is the regulation of mRNA stability through mixed A/G tailing (Figure 4) (Lim et al., 2018). TENT4 ncPAPs have promiscuous nucleotide specificity. Although ATP is their predominant substrate, they could also incorporate non‐adenosine residues, with the strongest preference for guanosine triphosphate over UTP and cytidine triphosphate. TENT4 ncPAPs incorporate single guanosine residues along with adenosines while extending long poly(A) tails of mRNAs, protecting them from the deadenylating complex CCR4‐NOT, because this complex is unable to efficiently remove the guanidine residue (Lim et al., 2018). The depletion of either *TENT4A* or *TENT4B* does not affect guanylation, whereas the depletion of both led to its strong decrease, suggesting that TENT4A and TENT4B are redundantly responsible for these additions (Lim et al., 2018). However, the ways in which TENT4A and TENT4B select their mRNA targets, presumably in the nucleus, remain to be established. Consistent with the potential stabilizing role of TENT4A/B is the observation that TENT4A and TENT4B stabilize viral RNA that is transcribed from hepatitis B virus (HBV) and human cytomegalovirus (HCMV) through the mixed tailing of their poly(A) (Hyrina et al., 2019; Kim et al., 2020; Mueller et al., 2019). This process occurs independently of other components of the TRAMP complex (i.e., zinc finger CCHC domain‐containing protein 7 [ZCCHC7] and hMTR4). Instead, TENT4A/B forms a complex with ZCCHC14 (Hyrina et al., 2019; Kim et al., 2020). Transcripts of both HBV and HCMV carry the posttranscriptional regulatory element (PRE). ZCCHC14 recognizes and binds to the CNGGN pentaloop within the PRE of viral transcripts and recruits TENT4A/B (Kim et al., 2020). Mixed tailing prevents the rapid deadenylation of viral RNA and results in its stabilization (Kim et al., 2020).

*Drosophila* has two homologs of yeast Trf4/5 ncPAPs that are expressed with the same pattern during development (Nakamura et al., 2008). Although both Trf4‐1 and Trf4‐2 bear the NTase domain, only Trf4‐1 has PAP activity in vitro. The knockdown or overexpression of *trf4‐1* is lethal, whereas the disruption of *trf4‐2* expression has no effect on the fly's viability. Trf4‐1, together with dmRrp6, participates in the polyadenylation‐mediated degradation of snRNAs in the nucleus (Nakamura et al., 2008). Additionally, Trf4‐1 has been proposed to facilitate exosomal 3′ mRNA decay in the cytoplasm (Harnisch et al., 2016).

In *C. elegans*, GLD‐4 is an ortholog of human TENT4B and TENT4A but appears to functionally diverge from these ncPAPs. GLD‐4 is expressed predominantly in the germline and localizes to the cytoplasm and P granules where its PAP activity relies on interactions with germline survival defective 1 (GLS‐1), an ortholog of human ZCCHC14 (Minasaki, Rudel, & Eckmann, 2014; Schmid et al., 2009). GLD‐4 is implicated in the regulation of some aspects of germline development through the cytoplasmic polyadenylation of mRNAs. However, in contrast to GLD‐2, the impact of GLD‐4 on bulk polyadenylation in the germline is mild, in which worms that lack *gld‐4* exhibit only minor changes in mRNA poly(A) tail length (Nousch et al., 2014). The depletion of *gld‐4* leads to no alterations of mRNA abundance, suggesting that GLD‐4 neither stabilizes nor promotes the degradation of mRNAs (Nousch et al., 2014). However, *gld‐4* mutant worms exhibit fewer polysomes, and both GLD‐4 and GLS‐1 proteins co‐purify with them (Nousch et al., 2014). Based on these observations, the catalytic activity of GLD‐4 has been hypothesized to be required for efficient polysome formation. Alternatively, GLD‐4/GLS‐1 might assist with polysome formation independent of the enzymatic activity of GLD‐4 (Nousch et al., 2014). The role of GLD‐4 in nuclear RNA surveillance has not yet been assessed. However, the wide evolutionary aspects of this function and fewer polysomes in GLD‐4 mutants suggest that it may also be the case in worms.

In summary, multiple functions have been attributed to the TENT4 family of PAPs. Mechanistically, the best understood role is nuclear RNA surveillance with TENT4 as a part of the TRAMP complex that attracts the RNA‐degrading exosome complex. The stabilizing function of mixed tailing by TENT4A/B is a very intriguing possibility. However, more data are needed to understand this phenomenon and its role in mRNA metabolism.

### 2.3 TENT5 family

In mammals, TENT5, also known as family with sequence similarity 46 (FAM46), is a family of four highly similar (>40% overall sequence identity) proteins—TENT5A, TENT5B, TENT5C, and TENT5D—that are differentially expressed in tissues and organs (Kuchta et al., 2016). TENT5 proteins are conserved in metazoa, and variable numbers of *TENT5* homologs are present in the genome of all animals (Kuchta et al., 2016). TENT5 proteins have been shown to act as cytoplasmic ncPAPs in functional in vitro and in vivo studies (Hu et al., 2020; Mroczek et al., 2017). The recently solved structure of TENT5B in *Xenopus tropicalis* revealed that TENT5 proteins share a two‐domain architecture that is comparable to other TENTs, although with some distinguishable properties (Hu et al., 2020). The N‐terminal NTase and C‐terminal helical domain of TENT5B form a large cleft where the highly conserved hG[GS] pattern and three catalytic residues [DE]h[DE]h and h[DE]h are located. The N‐terminal catalytic domain of xtTENT5B is prominently larger and contains more secondary elements compared with other PAPs. TENT5 proteins have been proposed to have the largest catalytic domain among all known PAPs. Another interesting characteristic of TENT5 proteins is that their overall fold resembles prokaryotic PAP/CCA adding enzymes. However, this similarity is only limited to the protein fold and not reflected by TENT5 activity, in which TENT5 proteins do not have CCA‐adding activity in vitro (Hu et al., 2020). Importantly, human TENT5B has almost exclusive preference for ATP incorporation, suggesting that TENT5 proteins are highly unlikely to perform mixed RNA tailing. Biochemical analysis revealed that human TENT5B prefers substrate RNAs that are adenosine‐rich close to the very 3′ end, suggesting that TENT5 proteins preferentially act on already polyadenylated mRNAs (Hu et al., 2020). The functional relevance of cytoplasmic polyadenylation by TENT5 ncPAP is only beginning to emerge because it is a relatively newly discovered family of TENTs (Figure 4) (Kuchta et al., 2016). Notably, despite a high level of similarity between TENT5 paralogs, they may have distinct functions.

#### 2.3.1 TENT5A

In mice, TENT5A has strong expression in embryonic and adult tissues that undergo mineralization, suggesting its role in bone development and postnatal bone homeostasis (Diener et al., 2016). TENT5A‐deficient mice exhibit growth delay, a shorter body length, and severe skeletal abnormalities (Diener et al., 2016). Furthermore, variable number of tandem repeat polymorphisms in the second exon of the *TENT5A* gene are associated with a higher risk of large‐joint osteoarthritis in humans (Etokebe et al., 2015). Loss‐of‐function mutations of the human *TENT5A* gene have been reported in patients who suffer from an autosomal recessive form of osteogenesis imperfecta bone disease (Doyard et al., 2018). The mechanism of TENT5A function in bones is currently unknown. TENT5A has been reported to be a potential binding partner of SMAD proteins, which are important components of signaling pathways that are involved in bone development (Colland et al., 2004). However, the functional relevance of this potential interaction to bone development in mammals has not been assessed. Interestingly, TENT5A has been shown to interact with Smad1/Smad4 and regulate the bone morphogenetic protein signaling pathway during early differentiation in *Xenopus* embryos (Watanabe et al., 2018). TENT5A is expressed in the frog preplacodal ectoderm, a specialized region that differentiates into the anterior pituitary gland and sensory organs, and TENT5A knockdown causes abnormalities in eye formation and body pigmentation (Watanabe et al., 2018). TENT5A was previously shown to be expressed in ameloblast nuclei of tooth buds and potentially involved in the development of teeth and enamel production in mice (Etokebe et al., 2009). Moreover, TENT5A has been described as a negative regulator of leptin during a low‐calorie diet (Carayol et al., 2017). Leptin is a hormone that regulates energy balance by inhibiting hunger and diminishing fat storage in human adipocytes. *TENT5A* knockdown does not influence leptin gene expression but leads to the higher secretion of leptin (Carayol et al., 2017). Additionally, mutations of the *TENT5A* gene are associated with the human autosomal recessive eye disease retinis pigmentosa (Barragán et al., 2008; Lagali, Kakuk, Griesinger, Wong, & Ayyagari, 2002; Schulz, Goetz, Kaschkoetoe, & Weber, 2004). A variable number of tandem repeat polymorphism in *TENT5A* was connected with susceptibility to tuberculosis (Etokebe, Bulat‐Kardum, Munthe, Balen, & Dembic, 2014). The molecular mechanism of action of TENT5A in these processes and pathogenesis of these diseases is unknown.

#### 2.3.2 TENT5B

As mentioned above, the structure of xtTENT5B protein has been solved, and the biochemical properties of TENT5B have recently been reported (Hu et al., 2020). However, few studies have described the functional relevance of this ncPAP. TENT5B has been proposed to serve as a potential molecular marker that may be useful for the diagnosis and prognosis of refractory lupus nephritis (Benjachat et al., 2015). The expression level of *TENT5B* is downregulated in patients with prostate cancer, and overexpression of the *TENT5B* gene inhibits prostate cancer cell proliferation in vitro and tumor growth in vivo (Liang et al., 2018).

Large‐scale transcriptomic analyses revealed that *TENT5B* is specifically expressed in human embryonic stem cells (hESCs) and human induced pluripotent stem cells, and its level is sharply downregulated during differentiation (Hu et al., 2020). In hESCs, *TENT5B* knockout is lethal, whereas knockdown leads to a lower abundance of newly synthesized proteins and the severely compromised viability of hESCs. A tempting speculation is that TENT5B is involved in the cytoplasmic polyadenylation of mRNA in the early stage of human embryogenesis (Hu et al., 2020).

#### 2.3.3 TENT5C

*TENT5C* is one of the most frequently mutated genes in multiple myeloma (MM), which occurs in approximately 20% of all cases. MM is a malignant proliferation of bone marrow plasma cells, and the loss of TENT5C is associated with limited survival (Barbieri et al., 2016; Boyd et al., 2011; Chapman et al., 2011; Hu, Chen, & Wang, 2019; Lohr et al., 2014; Vikova et al., 2019; Walker et al., 2018). Further work revealed that TENT5C is a B‐cell lineage‐specific growth suppressor. Several MM cell lines that carried mutations of the *TENT5C* gene exhibited significantly lower growth and lower survival rates upon the expression of wild‐type *TENT5C* (Mroczek et al., 2017; Zhu et al., 2017). Primary B cells that were isolated from *TENT5C* knockout mice proliferated faster than B cells that were isolated from wild‐type animals (Mroczek et al., 2017). *TENT5C* overexpression decreased levels of the transcription factors interferon regulatory factor 4, CCAAT/enhancer‐binding protein, and Myc proto‐oncogene protein and upregulated the expression of immunoglobulin (Ig) light chain and genes that are involved in the unfolded protein response (Zhu et al., 2017). The most crucial observation is that the reintroduction of active *TENT5C* into MM cell lines leads to the broad polyadenylation and stabilization of mRNAs, with a strong preference for those that encode endoplasmic reticulum (ER)‐targeted secreted proteins (Mroczek et al., 2017). It appears that TENT5C plays a broad role in B cell biology. Cytoplasmic polyadenylation by TENT5C was recently shown to stimulate the humoral immune response (Bilska et al., 2020; Herrero et al., 2020). A global analysis of poly(A) tail distribution by nanopore direct RNA sequencing revealed that mRNAs that encode Igs carry shorter poly(A) tails and have lower steady‐state levels in B cells that are isolated from TENT5C knockout mice compared with wild‐type mice (Bilska et al., 2020). *TENT5C*‐deficient B cells exhibit a reduction of Ig mRNA and protein abundance and secrete fewer antibodies (Bilska et al., 2020; Herrero et al., 2020).

TENT5C is also associated with other tumors (Tanaka et al., 2017; Wan et al., 2017; Zhang, Yue, Jiang, Han, & Xin, 2017). TENT5C expression is significantly lower in hepatocellular carcinoma (HCC) than in normal liver tissue (Zhang et al., 2017). TENT5C has been reported to serve as a biomarker of HCC recurrence and mediate the antimetastatic effects of norcantharidin on HCC cells (Wan et al., 2017; Zhang et al., 2017). A significant reduction of *TENT5C* levels was observed in gastric cancer, in which low TENT5C levels were associated with a poor prognosis and a higher risk of metastasis or recurrence (Tanaka et al., 2017). TENT5C also acts as a tumor suppressor gene in squamous cell carcinoma of the lungs (Xia et al., 2018) and oral cells (Zhuang & Lu, 2018).

TENT5C has been shown to be strongly expressed in spermatids in the mouse testes and play an essential role in spermiogenesis (Zheng et al., 2019). *TENT5C* knockout in mice results in male sterility, characterized by the production of headless spermatozoa or spermatozoa with severely compromised fertilization ability (Zheng et al., 2019). The exact mechanism of TENT5C function during spermiogenesis is unknown.

#### 2.3.4 TENT5D

TENT5D has strong polyadenylation activity in vitro, but only little data are available on the functional relevance of TENT5D. It has been proposed to be a testis cancer antigen, in which the *TENT5D* gene has a restricted expression pattern in the normal testis but is aberrantly expressed in testicular cancer (Bettoni et al., 2009). *TENT5D* is also overexpressed in the cerebral cortex in a mouse model of autism spectrum disorder (Hamilton et al., 2011).

### 2.4 MTPAP (TENT6) and DmMTPAP

MTPAP is the only known TENT that acts in mitochondria. It has an N‐terminal mitochondrial targeting sequence (residues 1–37) and localizes exclusively to this organelle where it contributes to the regulation of gene expression via the polyadenylation of mitochondrial RNAs (Nagaike, Suzuki, Katoh, & Ueda, 2005; Tomecki, Dmochowska, Gewartowski, Dziembowski, & Stepien, 2004). Structural and biochemical analyses indicate that MTPAP has strong substrate preference for ATP and acts as a dimer. Although it is rather self‐sufficient for substrate recognition, some proteins can stimulate its activity (Bai, Srivastava, Chang, Manley, & Tong, 2011; Chujo et al., 2012; Lapkouski & Hällberg, 2015; Wang et al., 2014). For example, leucine‐rich pentatricopeptide repeat‐containing protein (LRPPRC) stimulates the polyadenylation activity of MTPAP on sense mitochondrial mRNAs (mt‐mRNAs). Upon LRPPRC silencing, mt‐mRNAs have shorter poly(A) tails (Bratic et al., 2011; Chujo et al., 2012; Pajak et al., 2019; Wilson et al., 2014). Other mitochondrial enzymes, such as the RNA helicase SUV3 and RNA exoribonuclease PNPase, cooperate with MTPAP to regulate RNA metabolism in mitochondria (Wang et al., 2014). *C. elegans* appears to lack an ortholog of MTPAP. None of its closest homologs have been shown to reside in worm mitochondria, with no information about polyadenylation in *C. elegans* mitochondria. In contrast, the *D. melanogaster* genome encodes an ortholog of human MTPAP, DmMTPAP (CG11418). Despite only 30% homology between human and fly proteins, their functions and some properties are similar (Bratic et al., 2016). In the fruit fly, DmMTPAP is essential for mitochondrial function, fly development, and survival (Bratic et al., 2016). In humans, MTPAP is responsible for the polyadenylation of mitochondrial tRNAs and mRNAs. In *D. melanogaster*, mitochondrial rRNAs (mt‐rRNAs) have been shown to carry poly(A) tails. However, their functional relevance is not entirely clear (Bratic et al., 2016).

**FIGURE 5:**
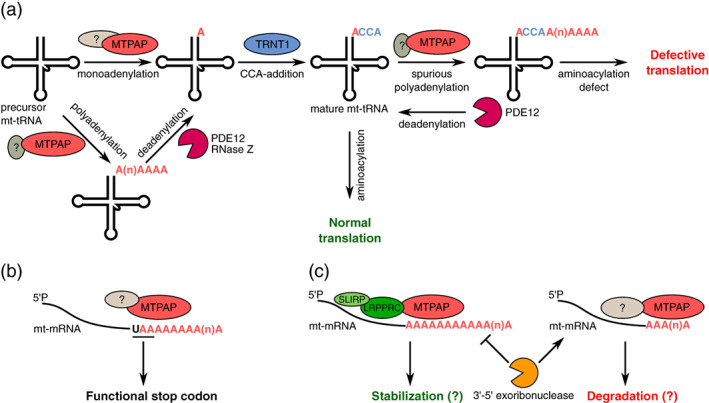
Mitochondrial poly(A) RNA polymerase (MTPAP) is the only TENT that acts in mitochondria. (a) MTPAP plays a role in the maturation of mt‐tRNAs through adenylation of the tRNA precursor, which generates a substrate for the addition of CCA and further acetylation. MTPAP also polyadenylates mature mt‐tRNAs, leading to defective translation. In both cases, the deadenylase PDE12 removes excessive adenosines. (b) MTPAP generates a complete UAA stop codon and functional ORFs for mt‐mRNA, for which full termination codons are not encoded in the mitochondrial genome. (c) The effect of polyadenylation on mt‐mRNA stability and turnover may be cofactor‐ and/or transcript‐dependent and is not completely understood. In humans and flies, the leucine‐rich pentatricopeptide‐repeat containing protein (LRPPRC)/stem‐loop‐interacting RNA‐binding protein (SLIRP) complex stimulates the polyadenylation activity of MTPAP and protects mRNA from degradation

MTPAP is important for the processing and maturation of mt‐tRNAs in both humans and flies (Figure 5a). In human mitochondria, the genes that encode tRNA^Tyr^ and tRNA^Cys^ overlap by one nucleotide that results in a tRNA^Tyr^ precursor that is devoid of 3′‐terminal adenosines. MTPAP is able to extend the tRNA^Tyr^ transcript 3′ end with a few adenosines. In the case of monoadenylation, the tRNA^Tyr^ precursor immediately becomes a substrate for the addition of CCA and further acetylation. If more adenosines are present, then either the deadenylase 2′,5′‐phosphodiesterase 12 (PDE12) or endonuclease RNase Z removes the excessive nucleotides, producing a substrate for the addition of CCA (Fiedler, Rossmanith, Wahle, & Rammelt, 2015; Pearce et al., 2017). Interestingly, in addition to the maturation of these two tRNAs, a spurious poly(A) tail could also be added by MTPAP to all other mt‐tRNAs, thus preventing their aminoacylation and leading to mitochondrial ribosome stalling and consequently a defect of the oxidative phosphorylation (OXPHOS) complex. PDE12 has been proposed to remove the spurious polyadenylation of mt‐tRNAs and restore the properly matured pool of mt‐tRNAs that are available for aminoacylation and correct translation (Pearce et al., 2017). In *D. melanogaster*, although genes that encode these tRNAs do not overlap, the loss of *dmmtpap* results in disturbances of maturation, a reduction of steady‐state levels of tRNA^Cys^, and incorrect CCA modification, indicating that polyadenylation by MTPAP is involved in the processing or stability of at least some mt‐tRNAs in flies (Bratic et al., 2016).

In humans, 7 of 13 mitochondrial genome‐encoded mRNAs (ND1, ND2, ND3, ND4, CytB, COIII, and ATP6) have incomplete stop codons with either a terminal U or UA because full termination codons are not encoded in the mitochondrial genome. For these mt‐mRNAs, adenylation by MTPAP is essential to generate a complete UAA stop codon and thus functional ORFs (Figure 5b) (Anderson et al., 1981; for review, see Borowski, Szczesny, Brzezniak, & Stepien, 2010; Gagliardi, Stepien, Temperley, Lightowlers, & Chrzanowska‐Lightowlers, 2004; Nagaike, Suzuki, & Ueda, 2008). Importantly, these mt‐mRNAs are essential, and in the case of MTPAP deficiency, these mt‐mRNAs would encode defective proteins that are unable to integrate into the OXPHOS complex. Apart from the generation of complete stop codons of mt‐mRNAs, MTPAP also polyadenylates mature mRNAs in mitochondria (Tomecki et al., 2004). The effect of poly(A) tails on mt‐mRNA stability, turnover, and translation has been a topic of discussion (for review, see Borowski et al., 2010; Gagliardi et al., 2004; Nagaike et al., 2008; Wilson et al., 2014). The downregulation of MTPAP leads to the shortening of mt‐mRNA poly(A) tails but also results in diverse effects on mt‐mRNAs, with some transcripts that are upregulated, some that are unaffected, and some that are downregulated (Nagaike et al., 2005; Rorbach, Nicholls, & Minczuk, 2011; Tomecki et al., 2004). These results suggest that the effects of MTPAP are mt‐mRNA‐specific, and individual transcripts may be differentially regulated, likely through additional factors (Figure 5c).

In fruit fly larvae, deletion of the *dmmtpap* gene leads to a severe reduction of mitochondrial polyadenylation, with all mt‐mRNAs having trimmed 3′ ends (Bratic et al., 2016). Surprisingly, despite the lack of functioning 3′ ends of mt‐mRNAs, these mRNAs were translated but led to the production of incomplete and defective proteins that could not be integrated into the correct complexes (Bratic et al., 2016). Interestingly, the loss of SUV3, PNPase, or MTPAP leads to the accumulation of mitochondrion‐derived antisense double‐stranded RNA in the cytoplasm of cells, which is associated with alterations of the immune response in flies (Pajak et al., 2019). However, whether polyadenylation is required for the degradation of these antisense RNAs and other RNA species in *D. melanogaster* remains to be established.

MTPAP has also been implicated in mitophagy, a process that involves the selective autophagic degradation of defective or damaged mitochondria (Furuya et al., 2018). The autophagy receptor NDP52 enters depolarized mitochondria and, together with MTPAP, forms an oligomeric autophagy receptor complex that presumably detects damaged mitochondria and facilitates their elimination. The MTPAP‐NDP52 interaction itself and not the catalytic activity of MTPAP has been proposed to be important for mitophagy (Furuya et al., 2018).

The disruption of RNA polyadenylation in human mitochondria that is caused by mutations of MTPAP has been linked to some genetic disorders. Notably, all disease‐related mutations have been identified within the fingers domain, which is a highly conserved region of MTPAP. Importantly, none of the identified mutations appears to affect the expression, oligomeric state, or cellular localization of MTPAP. The N478D mutation of MTPAP is associated with an autosomal‐recessive disease (i.e., severe progressive spastic ataxia with optic atrophy (Crosby et al., 2010). In individuals who carry the homozygous N478D mutation, the poly(A) tails of mt‐mRNAs are shorter compared with healthy individuals. Although the effect of tail shortening on mt‐mRNA stability is transcript‐dependent, it leads to alterations of mitochondrial protein expression and an OXPHOS defect (Wilson et al., 2014). The same homozygous N478D mutation causes a distinct cellular phenotype that is characterized by DNA damage, impairments in DNA repair kinetics, and an increase in cell death following exposure to ionizing radiation (Martin et al., 2014). Other rare missense mutations of MTPAP (i.e., heterozygous I428T and R523W mutations and the homozygous I385F mutation) have been associated with autosomal recessive perinatal lethal encephalopathy (Van Eyck et al., 2019). For N478D, these mutations also lead to a decrease in the polyadenylation of mitochondrial transcripts and alterations of the expression of mitochondrially encoded proteins.

## 3 URIDYLATION

RNA uridylation, although discovered much later than noncanonical polyadenylation, is a widespread posttranscriptional modification that targets various coding and noncoding RNAs in mammals, plants, trypanosomes, fission yeast, worms, fruit flies, and other organisms. TENT1/TUT1, TUT4/TENT3A, and TUT7/TENT3B are mammalian TUTases that mediate template‐independent uridylation. The functional ortholog of TENT1 in worms is USIP‐1, but its counterpart in flies is unknown. Tailor is a TUTase in fruit flies, which has functions that are reminiscent of TUT4/7 (Bortolamiol‐Becet et al., 2015). Worms also have two orthologs of TUT4/7, also known as poly(U) polymerases (PUPs): CDE‐1 (also known as PUP‐1 and CID‐1) and its paralog PUP‐2. The third TUTase, PUP‐3, is only distantly related to TUT4/7 (Jae & Wickens, 2007). *C. elegans* also has MUT‐2, the first enzyme that was shown to have the ability to add UG tails. This section summarizes our current knowledge about all known metazoan enzymes that drive RNA uridylation.

### 3.1 TENT1 (TUT1) and USIP‐1

In mammals, TENT1 is widely expressed in all tissues and essential for cell proliferation and survival (Hart et al., 2015; Trippe et al., 2006). TENT1 has a complex structure that consists of different domains that are distinct from those that are present in other TENTs. It comprises an N‐terminal zinc finger (ZF), an RNA‐recognition motif (RRM) followed by a palm domain that contains a proline‐rich region, fingers, a kinase‐associated‐1 (KA‐1) domain, and a C‐terminal nuclear localization signal (Yamashita, Takagi, Nagaike, & Tomita, 2017). Notably, TENT1 is the only member of the TENT family that carries the RRM and possibly does not rely on other RNA‐binding proteins for substrate specificity. TENT1 is localized to the nucleolus, nucleoplasm, and nuclear speckles (Mellman et al., 2008; Trippe et al., 2006).

**FIGURE 6:**
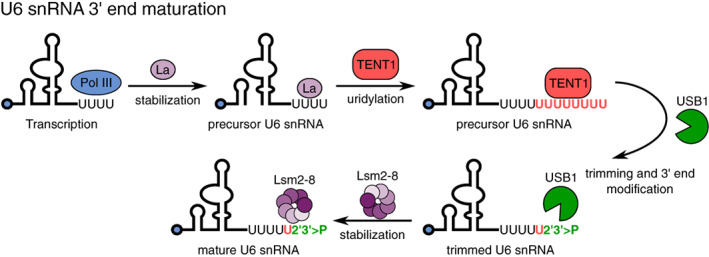
Maturation of U6 snRNA. Following transcription by Pol III, La protein protects the U6 snRNA precursor by binding the four uridines at the 3′ end. La protein is later replaced by TENT1, which uridylates the U6 snRNA 3′ end. The 3′‐5′ exoribonuclease and phosphodiesterase USB1 removes uridines, leaving only five of them, and a terminal 2′,3′ cyclic phosphate (2′3′ > P). The U6 snRNA is further protected by recruitment of the Lsm2‐8 protein complex. The blue dot at the 5′ end represents the γ‐monomethylguanosine triphosphate (meGTP) cap

Uridylation by TENT1 plays an essential role in the maturation of spliceosomal U6 snRNA that is responsible for the catalysis of pre‐mRNA splicing (Figure 6) (Hirai, Lee, Natori, & Sekimizu, 1988; Trippe et al., 2006; Trippe, Richly, & Benecke, 2003). U6 snRNA is transcribed by Pol III, which uses a short stretch of thymine nucleotides in DNA as a termination signal that results in four encoded uridines at the 3′ end of the U6 transcript (Lund & Dahlberg, 1992). Terminal uridines attract the chaperone‐like La protein, which stabilizes the U6 precursor (Pannone, Xue, & Wolin, 1998). To become fully functional, U6 snRNA undergoes further 3′ end maturation that starts with the removal of La protein and oligouridylation by TENT1 (Hirai et al., 1988; Trippe et al., 2003, 2006). Oligo(U) tails are subsequently trimmed by the 3′–5′ exoribonuclease USB1 until the tail contains only five uridines (Didychuk et al., 2017; Mroczek et al., 2012; Shchepachev, Wischnewski, Missiaglia, Soneson, & Azzalin, 2012; Shchepachev, Wischnewski, Soneson, Arnold, & Azzalin, 2015). USB1 also has very specific exoribonuclease activity that generates a 2′,3′‐cyclic phosphate at the RNA 3′ end—a hallmark of U6 snRNA that has a stabilizing effect by protecting it from oligouridylation‐mediated decay (Didychuk et al., 2017; Lund & Dahlberg, 1992; Mroczek et al., 2012; Shchepachev et al., 2012, 2015). The uridylation of U6 snRNA also plays an indirect important role in pre‐mRNA splicing by recruiting the LSM2‐8 protein complex to the 3′ end of U6 snRNA that facilitates the annealing of U6 snRNA with U4 snRNA and formation of the snRNP complex that is crucial for the splicing reaction (for review, see Didychuk, Butcher, & Brow, 2018).

The function of TENT1 is highly conserved among vertebrates, with some preservation of the degree of functional conservation among invertebrates. Similar to humans, U6 snRNAs in worms have four or more uridines at the 3′ end (depending on the *U6* snRNA gene isoform, in which four to five uridines are encoded in the DNA), and these ends are also protected at their 3′ termini. Intriguingly, the identity of the protective moiety/modification remains unknown, in which U6 snRNAs in worms do not carry 2′,3′‐cyclic‐, 3′‐monophosphate, or 2′‐*O*‐methylation blocking groups (Lund & Dahlberg, 1992; Rüegger, Miki, Hess, & Großhans, 2015). In *C. elegans*, the closest ortholog of TENT1 is USIP‐1 (U6 snRNA‐interacting protein‐1, ZK863.4), which is involved in regulating U6 snRNA stability and function (Rüegger et al., 2015). In *usip‐1*‐deficient mutants, the level of U6 snRNA is significantly decreased, in which molecules that have 3′ ends that cannot be extended to their normal 4–5 uridines are rapidly degraded (Rüegger et al., 2015). However, unclear is whether USIP‐1 directly participates in U6 snRNA biogenesis and stabilization because USIP‐1 interacts both physically and genetically with the bona fide U6 snRNA recycling factor squamous cell carcinoma antigen recognized by T‐cells 3.

There is some controversy about the substrate preference and function of TENT1. It was reported to function as a highly processive nuclear ncPAP called speckle targeted phosphatidylinositol‐4,5‐bisphosphate regulated PAP (Star‐PAP) (Mellman et al., 2008). Star‐PAP has been proposed to affect the expression of oxidative stress and apoptotic response genes by regulating the 3′ end formation of their mRNAs (Laishram & Anderson, 2010; Li et al., 2012; Li, Laishram, & Anderson, 2013; Mellman et al., 2008). Such a duality of TENT1 action could be explained by TENT1 substrate specificity and activity that are tightly regulated by its subnuclear localization, the presence of specific signals in RNA substrates, or the regulation of TENT1 through posttranslational modifications. Importantly, however, recent structural and biochemical studies revealed higher preference of TENT1 for uridylation over adenylation (Yamashita et al., 2017).

### 3.2 TUT4/7, CDE‐1, PUP‐2, PUP‐3, and Tailor

**FIGURE 7:**
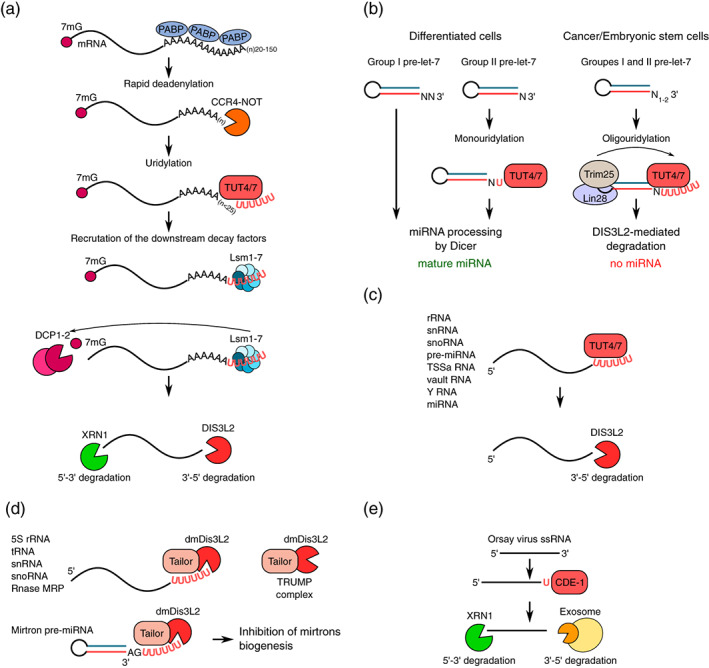
Selected uridylation‐dependent processes in humans, worms, and flies. (a) Uridylation‐mediated degradation of polyadenylated mRNAs in humans. After deadenylation, mRNAs with poly(A) tails that are shorter than 25 nt are uridylated by TUT4/7, leading to the recruitment of downstream RNA decay factors. The LSM1‐7 complex first interacts with the U‐tail and promotes mRNA decapping by the DCP1‐2 complex. mRNAs that are devoid of a cap are then degraded by the 5′–3′ exonuclease XRN1. Additionally, mRNA can be degraded by the exosome and/or DIS3L2 exonuclease in the 3′‐5′ direction. (b) Pre‐miRNA let‐7 uridylation by TUT4/7. Following their transcription and initial processing, the precursor miRNA (pre‐miRNA) are exported into the cytoplasm (not shown). In differentiated cells where Lin28 is absent, group II pre‐miRNAs that carry a one‐nucleotide 3′ overhang are monouridylated by TUT4/7, thus enabling further processing by Dicer. In cancer and embryonic stem cells, Lin28 binds both groups of let‐7 pre‐mRNAs and recruits TUT4/7. Dicer cannot process oligouridylated pre‐miRNAs, and they are degraded by the 3′‐5′ exonuclease DIS3L2. (c) Various RNA species that are transcribed by Pol I, Pol II, and Pol III can be targeted to the 3′–5′ exosome and/or DIS3L2 exonuclease degradation in a uridylation‐mediated manner. (d) In *Drosophila*, the decay of multiple RNAs relies on uridylation by Tailor, which forms a complex with the 3′–5′ exonuclease dmDis3L2 (a so‐called TRUMP complex). The stability of mirtrons is particularly controlled by uridylation. Mirtrons arise during splicing and lariat debranching of introns and carry AG at their 3′ ends. The 3′‐AG is uridylated by Tailor, which inhibits their biogenesis and leads to degradation by dmDis3L2. (e) Antiviral RNA uridylation in worms. Upon infection, Orsay virus RNAs are monouridylated by CDE‐1, which promotes their degradation by the 5′–3′ exonuclease XRN1 and 3′–5′ exonucleases of the exosome

Human TUT4 and TUT7 and their homologs are major mediators of uridylation that target a wide variety of coding and noncoding RNAs and RNA decay intermediates (De Almeida et al., 2018; Menezes et al., 2018). Uridylation is mostly linked to RNA surveillance and decay. However, depending on the substrate RNAs, cofactors, and the cellular context, uridylation may also be a crucial step of RNA biogenesis and maturation (Table 1 and Figure 7). TUT4 and TUT7 are proposed to act redundantly, and their functions are almost always described together, referring to them as TUT4/7. Human TUT4/7 and worm CDE‐1 are large multidomain enzymes, whereas other worm TUTases, such as PUP‐2 and PUP‐3, and the *D. melanogaster* TUTase Tailor are smaller and less complex (Figure 1).

Uridylation has been studied in great detail in the context of pre‐miRNA processing in both worms and humans (Figure 7b). In *C. elegans*, let‐7 miRNA regulates several transcription factors in the progression from larvae to adults; thus, its expression is tightly controlled to ensure a proper transition (Pasquinelli et al., 2000; Reinhart et al., 2000). The conserved RNA‐binding protein LIN‐28 binds pre‐let‐7, preventing its processing by Dicer. PUP‐2 uridylates pre‐let‐7 in a LIN‐28‐dependent manner, tagging it for degradation (Lehrbach et al., 2009). The mechanism is conserved in other species. In humans, the let‐7 miRNA family suppresses cell proliferation and promotes differentiation (Lee, Han, Kwon, & Lee, 2016). Furthermore, in differentiated cells, let‐7 suppresses several oncogenes and thus is repressed in some cancers (Balzeau, Menezes, Cao, & Hagan, 2017; Menezes et al., 2018). TUT4/7 plays a dual role in controlling let‐7 levels, either promoting the maturation of pre‐let‐7 or marking it for degradation. In humans, only 3 of 12 members of the let‐7 family carry the typical two‐nucleotide 3′ overhang in their precursors (group I pre‐miRNAs) and are directly processed by Dicer, whereas the rest have one nucleotide 3′ overhang (group II pre‐miRNAs) and require an additional step for their maturation (Bartel, 2018). In differentiated cells, monouridylation by TUT4/7 restores the two‐nucleotide 3′ overhang of group II pre‐let‐7, making them a substrate for Dicer and leading to effective let‐7 maturation (Heo et al., 2012; Kim et al., 2015). Importantly, this distributive activity of TUT4/7 at least partially arises from the absence of LIN28a in differentiated cells because LIN28a is a TUT4/7 positive processivity factor (Faehnle, Walleshauser, & Joshua‐Tor, 2017). In nondifferentiated cells and some cancers, LIN28a blocks let‐7 biogenesis to ensure pluripotency and self‐renewal (Hagan, Piskounova, & Gregory, 2009; Heo et al., 2009; Thornton, Chang, Piskounova, & Gregory, 2012). In this case, TUT4/7 oligouridylates pre‐let‐7, leading to its degradation by DIS3L2 (Faehnle, Walleshauser, & Joshua‐Tor, 2014; Hagan et al., 2009; Heo et al., 2009; Piskounova et al., 2011; Ustianenko et al., 2013, 2018). In addition to LIN28a, the E3 ligase tripartite motif‐containing protein 25 (Trim25) may stimulate the TUT4‐mediated oligouridylation of pre‐let‐7 (Choudhury et al., 2014).

In *D. melanogaster*, TUTase Tailor prevents the processing and promotes the degradation of mirtrons (Figure 7d) (Bortolamiol‐Becet et al., 2015; Reimão‐Pinto et al., 2015). Mirtrons are miRNA precursors that are located in introns of mRNA‐coding genes. Mirtrons rely on splicing and intron‐debranching machinery to generate short hairpins that end in AG, which are later processed by Dicer to produce mature miRNAs (Westholm & Lai, 2011). In fruit flies, mirtrons with AG‐ending hairpins are a preferred substrate for Tailor (Cheng et al., 2019; Kroupova, Ivascul, Reimao‐Pinto, Ameres, & Jinek, 2019). Uridylation promotes their degradation by dmDis3L2 and prevents the accumulation of mature miRNAs (Bortolamiol‐Becet et al., 2015; Reimão‐Pinto et al., 2015). Although mirtrons are also present in worms and humans, unknown is whether uridylation is involved in their decay.

Unclear are whether and how uridylation influences the activity and abundance of mature miRNAs. TUT4/7 has been reported to participate in the uridylation‐mediated degradation of mature miRNAs during differentiation and in response to environmental changes (Gutiérrez‐Vázquez et al., 2017; Jones et al., 2012; Thornton et al., 2014). Uridylation by TUT4/7 can also modulate target recognition, enhancing the repertoire of mRNAs that are regulated by a particular miRNA (Yang et al., 2019).

TUT4/7 also targets other RNA species for DIS3L2‐mediated decay, including aberrant snRNA, snoRNA, rRNA, 7SL, tRNA, Y and vault RNA, and Pol II transcription start site‐associated short RNA that are generated from bidirectional promotors (Figure 7c) (Łabno et al., 2016; Pirouz, Du, Munafò, & Gregory, 2016; Pirouz, Munafò, Ebrahimi, Choe, & Gregory, 2019; Ustianenko et al., 2016). This function is conserved in flies. Tailor, together with dmDis3L2, forms the cytoplasmic terminal RNA uridylation‐mediated processing complex that mediates cytoplasmic quality control and the degradation of various Pol III transcripts, such as 5S rRNA, tRNA, snRNA, snoRNA, and RNase MRP (Figure 7d) (Reimão‐Pinto et al., 2016).

In humans, TUT4/7 also regulates the lifespan of mRNAs. The TUT4/7‐mediated uridylation of replication‐dependent histone mRNAs recruits exosome to carry their degradation (for review, see Marzluff & Koreski, 2017). These mRNAs carry a stabilizing 3′ stem‐loop instead of a poly(A) tail. At the end of the S‐phase or during the inhibition of replication, histone mRNAs are no longer needed and are rapidly degraded in the TUT7‐mediated process. Interestingly, the activity of TUT4 in this process appears to play only a minor role, providing a rare example that TUT4 and TUT7 do not always act together (Lackey, Welch, & Marzluff, 2016). In addition to histone mRNAs, uridylation also initially targets polyadenylated mRNAs (Chang et al., 2014; Lim et al., 2014; Rissland & Norbury, 2008). In the cytoplasm, the poly(A) tails of mRNAs gradually become shortened by deadenylases until they can no longer accommodate PABPC (less than 25 nt). Such unprotected, short poly(A) tails are selectively uridylated by TUT4/7, triggering mRNA degradation from both ends (Figure 7a) (Chang et al., 2014; Lim et al., 2014; Malecki et al., 2013). The same mechanism occurs for mRNAs that are targeted for nonsense‐mediated decay in eukaryotes (Kurosaki, Miyoshi, Myers, & Maquat, 2018). Recent work with a conditional knockout mouse model strongly suggests that the TUT4/7‐mediated regulation of mRNA stability is particularly important during gametogenesis and early embryonic development (Chang et al., 2018; Morgan et al., 2019, 2017). The deletion of *TUT4* and *TUT7* leads to infertility and alterations of the transcriptome of germ cells, whereas the effect on somatic cells is minimal. Interestingly, in *D. melanogaster*, mRNAs and their degradation intermediates appear to not be frequently uridylated, suggesting that, in contrast to mammals, uridylation may not be the major trigger for their decay (Chang et al., 2014).

Finally, TUT4/7 also plays a role in the restriction of human LINE‐1 retrotransposition by the uridylation‐dependent degradation of LINE‐1 mRNA (Warkocki, Krawczyk, et al., 2018). LINE‐1 is a group of abundant vertebrate retrotransposons that proliferate through a “copy and paste” mechanism (Faulkner & Garcia‐Perez, 2017). LINE‐1 proliferation involves transcription and reintegration into a new site within genomic DNA by a so‐called target‐primed reverse transcription (TPRT) mechanism, potentially leading to de novo mutations of the germline, neurons, and cancers (Beck, Garcia‐Perez, Badge, & Moran, 2011; Scott et al., 2016). The uridylation of LINE‐1 mRNA enhances its degradation and blocks the initiation of reverse transcription during TPRT (Warkocki, Krawczyk, et al., 2018).

In *C. elegans*, uridylation by CDE‐1 is important in the regulation of various processes. In embryos, CDE‐1 localizes to mitotic chromosomes, where it interacts with the RNA‐dependent RNA polymerase EGO‐1 to produce endogenous small‐interfering RNAs (endo‐siRNAs) that are incorporated into the Argonaute (Ago) protein CSR‐1. CDE‐1 uridylates these siRNAs to limit their accumulation (van Wolfswinkel et al., 2009). Worms that lack *cde‐1* exhibit an increase in endo‐siRNA levels and defects in both mitotic and meiotic chromosome segregation, likely because of the inappropriate loading of siRNAs into pathways that are mediated by other Ago proteins. Thus, CDE‐1 protects the transcriptome against excessive EGO‐1‐generated siRNAs and restricts them to the CSR‐1‐mediated RNAi pathway (van Wolfswinkel et al., 2009). A related process has been demonstrated in the fission yeast *Schizosaccharomyces pombe*, in which spurious small RNAs are eliminated to prevent the unintended silencing of euchromatin genes (Pisacane & Halic, 2017). CDE‐1 was also identified in the forward genetic screen as a gene that is required for RNAi inheritance, a phenomenon in which the progeny of animals that are exposed to double‐stranded RNA continue to silence genes that are targeted by these RNAs in previous generations (Spracklin et al., 2017). The role of CDE‐1 in long‐term gene silencing needs further investigation, but CDE‐1 likely uridylates siRNAs that are associated with the Ago protein WAGO‐4 that promotes the transmission of siRNAs for RNAi inheritance (Spracklin et al., 2017; Xu et al., 2018).

In *C. elegans*, the balance between CDE‐1, PUP‐2, and PUP‐3 activity is essential for germline development (Li & Maine, 2018). The lack of CDE‐1 and PUP‐2 allows the expression of soma‐specific genes in the germline, leading to severe defects in development and reproduction. Moreover, under temperature stress, *cde‐1*/*pup‐2* double‐mutant worms do not maintain the identity of the germline and exhibit transgenerational germ cell loss. However, the identity of direct targets of CDE‐1 and PUP‐2 is unknown. Interestingly, PUP‐3 levels increased in the *cde‐1*/*pup‐2* double‐mutant germline, indicating that CDE‐1 and PUP‐2 may regulate PUP‐3 levels. The loss of *pup‐3* partially rescues the germline phenotypes in the *cde‐1*/*pup‐2* mutant. PUP‐3 targets are most likely distinct from CDE‐1 and PUP‐2 substrates, and PUP‐3 likely exerts an opposite effect on germline development. The authors proposed that the coordinated activity of these three enzymes guarantees the proper abundance of their RNA targets, which impacts gene expression in the developing germline (Li & Maine, 2018).

CDE‐1 and human TUT4/7 play a critical role in antiviral immunity (Le Pen et al., 2018). In worms during infection with Orsay virus, CDE‐1 uridylates the 3′ end of the viral RNA genome and promotes its degradation (Figure 7e). The depletion of diverse RNA decay enzymes, such as those that are encoded by *dis‐3*, *exos‐2*, and *xrn‐2*, led to the accumulation of uridylated viral RNAs, indicating their contribution to viral RNA removal. Human TUT4/7 enzymes mark influenza A virus mRNAs for degradation, thus decreasing the rate of infection (Le Pen et al., 2018). In worms, the uridylation pathway acts in parallel with RNAi to fight RNA viruses. In mammals, the uridylation pathway appears to complement innate and adaptive immune responses. In other organisms, many exogenous RNAs of viral origin are oligouridylated, suggesting that uridylation is a conserved antiviral mechanism (Huo et al., 2016; Yeo & Kim, 2018).

### 3.3 MUT‐2

MUT‐2 (also known as RDE‐3) is a TENT that is homologous to GLD‐2, MTPAP, and CDE‐1. It was first identified in a screen of mutants with a higher frequency of transposon Tc1 excision in the *C. elegans* germline (Collins, Saari, & Anderson, 1988). Since then, it has been implicated in transposon silencing and the enhancement of RNAi (Chen et al., 2005; Gu et al., 2009; Tsai et al., 2015). The latest findings show that MUT‐2 can add long stretches that contain tandem UG repeats (pUG tails) both in vitro and when tethered to mRNA in heterologous expression systems (Preston et al., 2019). Furthermore, in vivo, MUT‐2 has been reported to add pUG tails to transposon RNAs and to other targets of RNAi (Shukla et al., 2020). Such extension leads to the recruitment of RNA‐dependent RNA polymerase that directly synthesizes siRNAs. In turn, these small RNAs help target MUT‐2 to its substrates, which creates a self‐reinforcing loop that enables RNA‐based transgenerational inheritance (Shukla et al., 2020).

## 4 OUTLOOK

As summarized in the present review, metazoan TENTs are crucial for homeostasis in animals through the regulation of gene expression at the posttranscriptional level. RNA tailing clearly participates in many biological processes, although we are only at the beginning of exploring its significance. Thus, much more needs to be discovered.

An important aspect of TENT function that is critical for further progress in the field is to understand the molecular mechanisms that underlie the specificity of TENT enzymes. TENTs often rely on interacting RNA‐binding proteins to identify their target mRNAs (Martin & Keller, 2007). In worms, GLD‐2 substrate selectivity is provided by interactions with the RNA‐binding proteins GLD‐3 and RNP‐8. Interestingly, the GLD‐2/GLD‐3 and GLD‐2/RNP‐8 complexes modify distinct pools of mRNAs and control gamete identity by promoting spermatogenesis or oogenesis, respectively (Kim et al., 2009, 2010; Nakel et al., 2016). In *Drosophila*, Wispy relies on the CPEB homolog Orb and other RNA‐binding proteins, such as Dicer‐2 and Aubergine, for target RNA recognition (Benoit et al., 2008; Coll et al., 2018; Cui et al., 2013; Dufourt et al., 2017). In *Xenopus*, cytoplasmic polyadenylation by TENT2 is CPEB‐dependent (for review, see Radford et al., 2008; Reyes & Ross, 2016). In mammals, the situation is more complicated. Although TENT2 knockout mice have no discernible phenotype, knockouts of CPEB family members result in a plethora of different phenotypes, including gametogenesis defects (Ivshina et al., 2014; Maillo et al., 2017). Thus, two alternative scenarios are possible. CPEBs may cooperate with other TENTs or CPEB performs biological functions that are not directly related to cytoplasmic polyadenylation. Further research is needed to resolve this issue. TENT4A/B acts in complex with other proteins (e.g., TRAMP) to target RNA for exosome‐mediated degradation (Fasken et al., 2011; LaCava et al., 2005) but can also be recruited to other substrates, such as viral RNAs, by ZCCHC14 (Hyrina et al., 2019; Kim et al., 2020). TUT4/7 substrate specificity is highly dependent on the molecular context. For the uridylation of mRNA, a reduction of poly(A) tail length to less than 20 nt appears to be a sufficient trigger (Lim et al., 2014). In the case of the LINE‐1 retrotransposon, this process requires cooperation with the helicase Moloney leukemia virus 10 (MOV10) (Warkocki, Krawczyk, et al., 2018). However, for other RNA species, TUT4/7 relies on many RNA binding cofactors and other target selectivity mechanisms (Choudhury et al., 2014; Faehnle et al., 2014; Hagan et al., 2009; Heo et al., 2009). Finally, MTPAP does not appear to require any interaction partner to polyadenylate mRNAs. The same is true for the uridylation of U6 snRNA by TENT1. For other TENT families, such as TENT5, the mechanism of substrate selection remains to be established.

Another issue is the nucleotide specificity and processivity of enzymes. Despite quite intensive efforts, the molecular mechanisms that underlie the selection of nucleotides by TENTs are not fully understood. Some explanations have been proposed for uridines over adenosines selection by TUTases, such as TUT7, Tailor, and lower eukaryotic counterparts (Kobylecki, Kuchta, Dziembowski, Ginalski, & Tomecki, 2017; Munoz‐Tello, Gabus, & Thore, 2012, 2014; Yates et al., 2012). Still unknown, however, is why TENT4 family members incorporate guanosines with relative frequency. The best way to predict specificity is currently based on overall sequence similarity with the best biochemically characterized members. However, there are peculiarities. For example, the worm NPOL‐1, paralogous to GLD‐2 and orthologous to human TENT2, has broad nucleotide specificity with a slight preference for uridines/adenosines (Preston et al., 2019) and does not promote mRNA translation once tethered to reporter mRNA, unlike its other family members (Jae & Wickens, 2007). The basis of TENT processivity and the switch between processive and distributive modes remain largely unknown. Some enzymes, such as TUT4/7, are processive in vitro for unstructured substrates and distributive for structured substrates (Faehnle et al., 2017; Warkocki, Krawczyk, et al., 2018). Based on biochemical experiments in TENT4 orthologs in yeast, the RNA helicase subunit of the TRAMP complex has been proposed to control the processivity of PAP (Jia et al., 2011). Although the exact mechanisms that modulate other TENT modes of action await further investigation, one hypothesis could be that structural properties of RNA substrates and TENT cofactors are important in this regard.

Finally, TENT activity is known to play an important role in some biological processes, such as the activation of dormant mRNAs during oogenesis, and in some neuronal processes. In mammals, however, the identity of the enzyme that is responsible for this process remains to be established.

## CONFLICT OF INTEREST

The authors have declared no conflicts of interest for this article.

## AUTHOR CONTRIBUTIONS

**Vladyslava Liudkovska:** Visualization; writing‐original draft; writing‐review and editing. **Andrzej Dziembowski:** Conceptualization; funding acquisition; supervision; writing‐original draft; writing‐review and editing.

## RELATED WIREs ARTICLES

Specificity factors in cytoplasmic polyadenylation

Deadenylation: Enzymes, regulation, and functional implications.

Poly(A) RNA‐binding proteins and polyadenosine RNA: New members and novel functions.

Exonucleases and endonucleases involved in polyadenylation‐assisted RNA decay

Control of poly(A) tail length
